# Effect of a *Bifidobacterium*-Containing Acid-Resistant Microcapsule Formulation on Gut Microbiota: A Pilot Study

**DOI:** 10.3390/nu14224829

**Published:** 2022-11-15

**Authors:** Miki Minami, Shoji Tsuji, Shohei Akagawa, Yuko Akagawa, Yuki Yoshimoto, Hirosato Kawakami, Mamiko Kohno, Kazunari Kaneko

**Affiliations:** 1 Department of Pediatrics, Kansai Medical University, Osaka 573-1010, Japan; 2 Research and Development Department, Morishita Jintan Co., Ltd., Osaka 573-0128, Japan

**Keywords:** acid-resistant microcapsule, *Bifidobacterium breve* M-16V, *Bifidobacterium longum* BB536, gut microbiota, pediatrics

## Abstract

Approximately 10 *Bifidobacterium* species are known to inhabit the human intestinal tract. Bifidobacteria have been reported to possess a variety of probiotic benefits. However, when bifidobacteria are consumed internally as probiotics, the bacteria are killed by gastric acid. Therefore, we developed acid-resistant microcapsules containing *Bifidobacterium breve* M-16V and *B. longum* BB536, which are unaffected by gastric acid, and evaluated whether the microcapsule formulation increased the amount of bifidobacteria in the stool after administration compared with the powder formulation. The results revealed no significant difference in the percentage or number of *B. longum* between before and after administration of the powder or microcapsule formulation in children. By contrast, the bacterial count of *B. breve* was significantly increased after microcapsule formulation administration (1.5 × 10^5^ copies/g after administration versus 2.8 × 10^4^ copies/g before administration, *p* = 0.013). In addition, the increase in the bacterial count of *B. breve* in stools after administration of microcapsule formulation was approximately 1000-fold higher than that after powder formulation administration (*p* = 0.018). In conclusion, the results indicate that the microcapsule formulation is efficiently transferred to the large intestine without the adverse effects of gastric acidity in children.

## 1. Introduction

The human oral cavity, gastrointestinal tract, respiratory tract, skin, and urinary and reproductive organs are inhabited by more than 1000 species of bacteria numbering approximately 38 trillion. The bacterial population in the gastrointestinal tract, which accounts for more than 90% of all inhabiting bacteria, comprises the gut microbiota and lives in symbiosis in the human gut, maintaining a certain balance [1]. The composition and quantity of the gut microbiota change with age: the gut microbiota of 1-year-old children is dominated by bacteria belonging to the Actinobacteria phylum; however, their abundance gradually declines, and by 3 years of age, bacteria belonging to the Firmicutes phylum are dominant, similar to the findings in adults [2]. Previously, evaluation of the gut microbiota was based on the classical method of bacterial culture, but with the advent of next-generation sequencers, it is now possible to perform comprehensive genetic analysis to evaluate bacteria, including those that have been difficult to cultivate in the past [3].

Dysbiosis describes abnormalities in the composition or quantity of the gut microbiota, and it is known that dysbiosis occurring from birth to about 2 years of age is transferred to adulthood [4,5]. It has been reported that dysbiosis occurring in infancy is associated with the subsequent development of chronic diseases such as allergic diseases, inflammatory bowel disease, autoimmune disease, obesity, and diabetes mellitus [6]. The American Academy of Pediatrics has stated that a child’s mental development and risk of diseases in childhood and adulthood, such as obesity, hypertension, and diabetes, are related to nutrition in the first 1000 days of life [7]. Therefore, methods for disease prevention and treatment that maintain a normal gut microbiota or correct dysbiosis are being considered. Probiotic administration is considered one such method. Since the first 1000 days of life coincides with the establishment of the gut microbiota, the use of probiotics from infancy should be considered for children with dysbiosis. Probiotics are live microorganisms that, when consumed in proper amounts, confer beneficial effects on the health of the host [8]. For probiotics to exert their functions, they must be sufficiently safe, be of human origin, be able to withstand gastric juice and bile and reach the intestine, be able to grow in the intestines, and be able to be taken orally and maintain an effective bacterial count in the gastrointestinal tract [8].

*Bifidobacterium* and *Lactobacillus* species are used as typical probiotics. Lactobacilli are facultative anaerobic bacteria found mainly in the small intestine and female reproductive organs (vagina, cervix, and uterus). Meanwhile, bifidobacteria are anaerobes that cannot grow in the presence of oxygen, and they are found mainly in the large intestine [9]. More than 50 species of *Bifidobacterium* have been identified [10]. Human residential bifidobacteria (HRB) are broadly classified into adult and infantile forms, with adult HRB including *Bifidobacterium adolescentis*, *B. pseudocatenulatum*, *B. angulatum*, and *B. dentium* and infantile HRB including *B. breve*, *B. longum* subsp. *infantis*, and *B. bifidum*. Among HRBs, only *B. longum* subsp. *longum* is found in a wide range of ages ranging from infants to older individuals [11]. Bifidobacteria have been reported to exhibit a variety of beneficial effects, including intestinal regulation, protection against infection, and suppression of allergic reactions [12,13]. Probiotics containing *B. infantis* reduced the incidence and severity of necrotizing enterocolitis in very-low-birth-weight infants [14,15]. *Bifidobacterium* treatment improved the atopic dermatitis score [16,17,18] and reduced IgE levels [19] in patients with atopic dermatitis.

Although the effects of bifidobacteria as probiotics are proven, it is reported that approximately 40% of the administered bifidobacteria are killed by stomach acid when consumed as a powder formulation [20]. Therefore, we developed acid-resistant microcapsules containing bifidobacteria to ensure the efficient delivery of the bacteria to the large intestine, where bifidobacteria exert their effects as probiotics. The microcapsule manufacturing technology possessed by the authors makes it possible to prepare nearly spherical microcapsules with diameters ranging from 0.5 to 8 mm, which are relatively smaller than conventional capsules. The microcapsules can be given properties such as acid resistance, heat resistance, and enteric solubility by adding multiple layers of film, and the range of formulation design is remarkably wide. The microcapsules used in this study, which are acid-resistant and enteric-soluble, can be used as a kind of drug delivery system to deliver functional ingredients such as bifidobacteria to the intestines without being altered by the low pH of gastric acid [21]. Using the above-mentioned manufacturing technology, a product containing only *B. longum* BB536 for adult use is marketed under the trade name “Bifina^TM^”.

In this study, we developed an acid-resistant microcapsule for children’s use containing *B. breve* M-16V and *B. longum* BB536 that is not expected to be affected by gastric acid. The purpose of this study was to evaluate whether the microcapsule formulation more efficiently reaches the intestinal tract after oral administration than the powder formulation.

## 2. Materials and Methods

### 2.1. Study Participants

Healthy children were recruited, and after explaining the study to their guardians, 10 children were enrolled after their guardians provided informed consent. The inclusion criteria for participants were age of 1–12 years, no use of antimicrobials in the last 3 months, and no regular visits to hospitals. The participants included eight boys and two girls with a median age of 8.08 years (interquartile range (IQR) = 5.83–9.44) on the study entry date. No study participants were born preterm or low-birth weight, which were considered to be risk factors for gut dysbiosis: the median week of gestation was 40.0 weeks (IQR = 39.3–40.1), and the median birth weight was 2950 g (IQR = 2791–3140).

### 2.2. Characteristics of the Acid-Resistant Microcapsule

**Figure 1:**
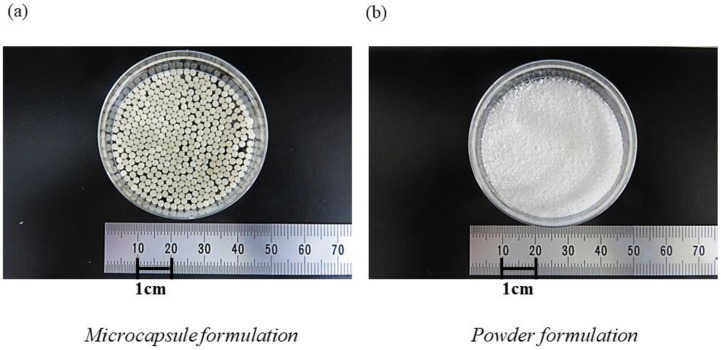
Appearance of formulation of used probiotics. The appearance of the bifidobacteria-containing formulations used in this study are shown: (**a**) the microcapsule formulation and (**b**) the powder formulation. Each microcapsule formulation was approximately 2 mm in size.

The acid-resistant microcapsule formulation administered to the study participants contained *B. breve* M-16V and *B. longum* BB536 at a ratio of 1:9 and a total of 1 billion bacteria per packet. The weight of one packet was 0.2 g. The acid-resistant microcapsule used in this study is a seamless, spherical microcapsule with a structure that prevents external liquids and other substances from entering through the outer membranes, as shown in Figure 1a. The microcapsule was constructed using the “drop-in-liquid” method, which is a manufacturing method that utilizes interfacial tension. The microcapsule has a three-layer structure prepared using a triple nozzle. The outermost layer of the membrane contains a substance (gelatin and pectin) that does not dissolve in the acidic range but does dissolve in the neutral range, and the pH response is designed so that the microcapsule does not dissolve in the stomach but instead dissolves in the intestine. The inner membrane of the middle layer of the capsule is made of vegetable fats and oils to improve the barrier function against the permeation of stomach acid. The innermost layer contains the contents (lyophilized *Bifidobacterium* powder), and a type of drug delivery system is realized by the aforementioned two-layer outer and middle membranes. Each microcapsule was designed to be 2 mm in diameter. A powdered formulation of *B. breve* M-16V containing 1 billion bacteria per packet was also produced as a control to confirm the effectiveness of the acid-resistant microcapsules (Figure 1b).

### 2.3. Evaluation of Gut Microbiota before and after the Oral Administration of Bifidobacteria-Containing Products

**Figure 2:**
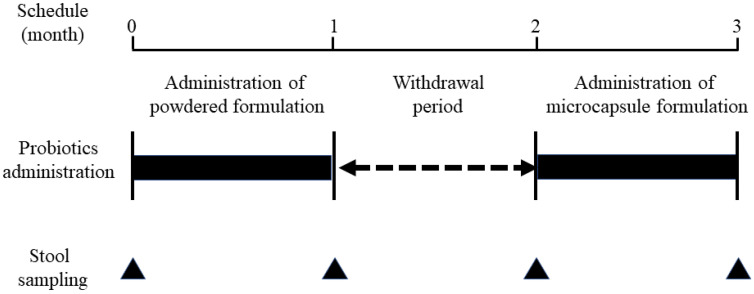
Study design Participants first received one packet of *B. breve* M-16V powder for one month; after a one-month rest period, a microcapsule formulation was administered orally (one packet per day) for one month. A powder formulation contained 1 billion bacteria per packet, while a microcapsule formulation contained *B. breve* M-16V and *B. longum* BB536 at a ratio of 1:9 and a total of 1 billion bacteria per packet.

Figure 2 illustrates the study design. Participants were first given one packet of *B. breve* M-16V powder per day for 1 month. After a 1-month withdrawal period, the microcapsule formulation was administered orally for 1 month (one packet per day). To assess study participants’ adherence to the formulation, a questionnaire regarding the powder or microcapsule formulation was administered to their parents. At the same time, information on the frequency of defecation and changes in stool characteristics was collected. No restrictions were placed on the intake of probiotics other than bifidobacteria. If any probiotics other than bifidobacteria were ingested, we asked the participants to indicate them on the questionnaire form.

To analyze the gut microbiota, spontaneously excreted stool samples of the study participants were collected 4 times before and after 1 month of powder or microcapsule consumption, as shown in Figure 2, and stored at −80 °C until DNA extraction.

### 2.4. Analysis of Gut Microbiota

For analysis of the gut microbiota, fecal bacterial 16S rRNA sequencing was performed to elucidate the changes in the taxonomic composition and alpha diversity of the study participants for each interventional period. In addition, the relative abundance of *B. longum* and *B. breve*. Additionally, quantitative analysis of the bacterial count of *B. breve* was performed by quantitative real-time polymerase chain reaction (RT-PCR).

### 2.5. Determination of the 16S rRNA Sequence

DNA extraction was performed using a NucleoSpin DNA Stool Kit (MACHEREY-NAGEL, Düren, Germany) within 1 week after stool collection. Seven hypervariable regions of the 16S rRNA regions of the extracted DNA, excluding v1 and v5, were amplified using a 16S Metagenome Kit (Thermo Fisher Scientific, Waltham, MA, USA). After purification, libraries were constructed using an Ion Plus Fragment Library Kit (Thermo Fisher Scientific) and Ion Xpress Barcode Adapters Kit (Thermo Fisher Scientific). The barcode libraries were quantified using an Agilent Bioanalyzer 2000 (Agilent, Santa Clara, CA, USA). The Ion Chef Instrument and Kit were used to obtain specific concentrations of emulsion PCR and templates. Sequence analysis was performed using an Ion GeneStudio S5 sequencer (Thermo Fisher Scientific) and Ion 318 chip (Thermo Fisher Scientific). Sequence data were analyzed using Metagenomics 16S w1.1 v5.16 workflow (Thermo Fisher Scientific) and Ion Reporter software.

MicroSEQ 16S Reference Library v2013.1, GreenGenes v13.5, Ion Reporter software, and Metagenomics 16S w1.1 v5.16 workflow (Thermo Fisher Scientific) were used to cluster the raw BAM files into operational classification units with 97% identity, and the data were analyzed using the default settings (read length filter, 150; minimum aligned cover age, 90.0; read abundance filter, 10; slash ID reporting rate, 0.2). Ion Reporter’s Quantitative Insights Into Microbial Ecology open-source bioinformatics pipeline was used for alpha and beta diversity analysis and visualization. Only data with a total read count of at least 5000 were used in the analysis.

### 2.6. Quantitative Measurement of B. breve in Stools Using PCR

RT-PCR targeting the groEL gene of *B. breve* was used to quantify *B. breve* in human stool [22]. Quantitative RT-PCR was contracted to PGL LLC (Aichi, Japan). The primer sequences used in RT-PCR were as follows: groEL gene of *B. breve* forward, GCTCGTCGTTGCCGCCGCCAAGGACGTT; groEL gene of *B. breve* reverse, ACAGAATGTACGGGATCCTCGAGCACG.

The PCR instrument was an Applied Biosystems 7500 Fast Real-Time PCR System (Thermo Fisher Scientific), and KOD SYBR^®^ qPCR Mix was used to generate reactions. The temperature program for PCR for groEL gene of *B. breve* was as follows: 98 °C for 2 min, followed by 40 cycles at 98 °C for 10 s, 60 °C for 10 s, and 68 °C for 34 s. The number of *B. breve* groEL genes in stool samples was calculated by plotting CT values obtained via 10-fold serial dilutions of quantitative PCR standards as a linear function of the base 10 logarithm of the known concentration to create a standard curve.

### 2.7. Statistical Analysis

Continuous variables were expressed as the median and IQR. The Wilcoxon signed-rank test was used for comparisons between the two corresponding groups. Statistical significance was set at *p* < 0.05. Statistical analysis was performed using BellCurve for Excel (version 3.21; Social Research and Information Center, Tokyo, Japan).

## 3. Results

### 3.1. Characteristics of the Study Participants

Table 1 summarizes the participants’ adherence to the probiotic formulation, the frequency of defecation, and changes in stool characteristics associated with the administration of the probiotic formulation.

Study participants’ adherence to the microcapsule formulation was good, with a median rate of 100% (IQR = 96.6–100). The stool frequency after 1 month of oral administration of the powder or microcapsule formulation was checked. There was no statistically significant difference in the median stool frequency per week between before and after powder formulation administration (5.0 times/week [IQR] 4.5–6.0] vs. 5.75 times/week [IQR = 5.0–6.0], *p* = 0.35). Similarly, the stool frequency did not differ between before and after oral administration of the microcapsule formulation (5.75 times/week [IQR = 5.0–6.0] vs. 3.25 times/week [IQR = 2.75–5.75], *p* = 0.18).

Differences in the Bristol Stool Form Scale (BSFS) score [23] between before and after oral administration of the powder or microcapsule formulation were evaluated. The results revealed no statistically significant difference, with a median BSFS score of 4.0 (IQR = 3.0–4.0) after 1 month of powder administration compared with 4.0 (IQR = 3.0–4.0) before oral administration (*p* = 0.56). Additionally, the median BSFS score after microcapsule formulation administration was 4.0 (IQR = 4.0–4.0) versus 4.0 (IQR = 3.0–4.0) before administration (*p* = 0.32).

### 3.2. Results of Gut Microbiota Analysis

#### 3.2.1. Taxonomic Composition

**Figure 3:**
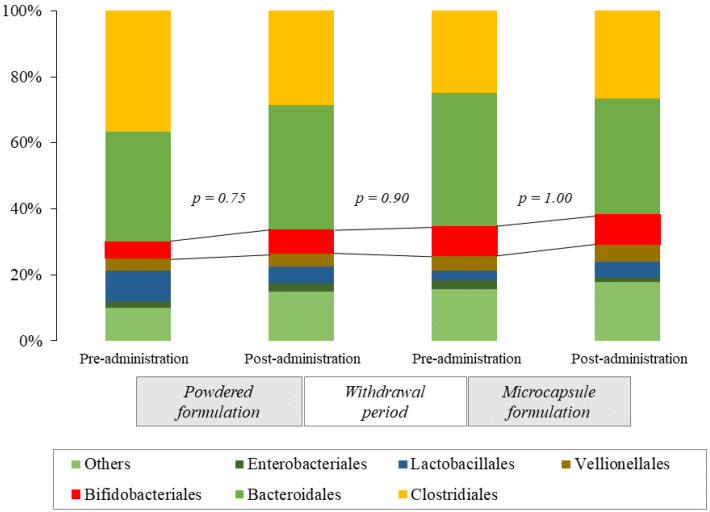
Changes in the composition of the gut microbiota after oral administration of bifidobacterial formulations at the order level. Bars represent the averages of individual data. The percentage of Bifidobacteriales (red bars) did not show any statistically significant change before or after administration of the *Bifidobacterium*-containing powder and microcapsule formulations.

The composition of the gut microbiotas of the study participants for each intervention period is shown in Figure 3. At the order level, the percentage of Bifidobacteriales did not change before or after oral administration of *Bifidobacterium*-containing powders or microcapsules.

#### 3.2.2. Alpha Diversity

**Figure 4:**
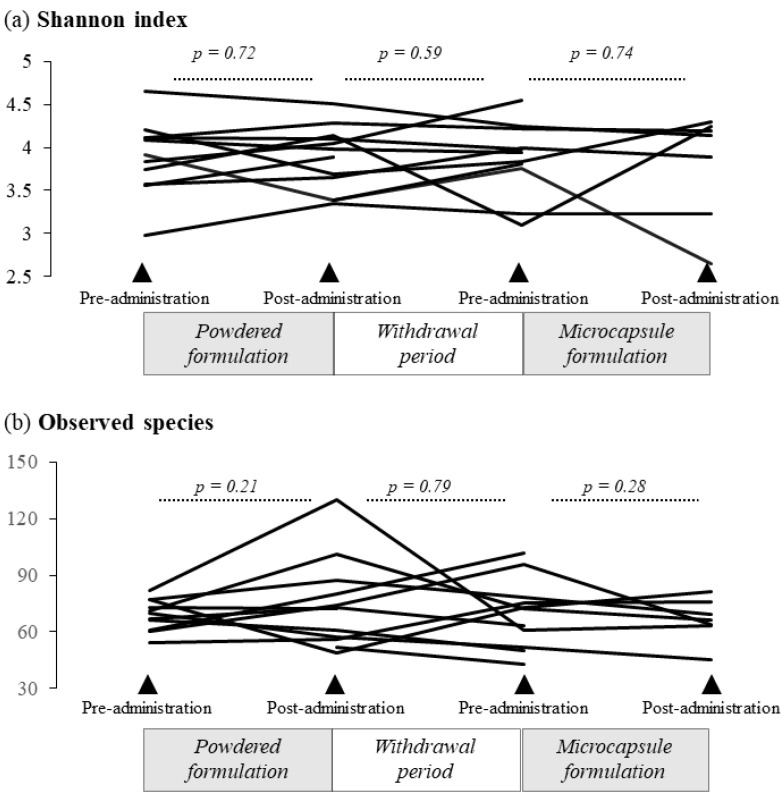
Changes in the Shannon index and observed species of the gut microbiota after administration of the powder or microcapsule formulation containing bifidobacteria. (**a**) There was no statistically significant difference in the Shannon index representing alpha diversity before or after administration of the powder, the withdrawal period, or administration of the acid-resistant microcapsule formulation containing bifidobacteria. (**b**) The observed species also displayed no statistically significant difference in abundance after administration.

Figure 4a presents the results of the Shannon index and observed species of gut microbiota. The Shannon index was 3.98 (median; IQR = 3.67–4.12) after powder formulation administration, compared with 3.91 (median; IQR = 3.66–4.11) before administration (*p* = 0.72). There was no significant difference in the Shannon index during the one-month withdrawal period (*p* = 0.59). Similarly, there was no significant difference in this index before (median 3.83; IQR = 3.49–4.11) and after microcapsule formulation administration (median 4.14; IQR = 3.56–4.21), *p* = 0.74). Meanwhile, Figure 4b shows the number of observed species of gut microbiota. In total, 73 species (median; IQR = 59–83.5) were observed after powder formulation administration, compared with 70 species (median; IQR = 63–75) before treatment (*p* = 0.21). There was no significant difference in the change in the observed species during the one-month withdrawal period (*p* = 0.79). Meanwhile, 73 species (median; IQR = 66.5–76.5) were detected before microcapsule formulation administration versus 66 species (median; IQR = 63.5–72.5) after administration (*p* = 0.28).

#### 3.2.3. Relative Abundance of *B. longum* and *B. breve* in the Gut Microbiota

**Figure 5:**
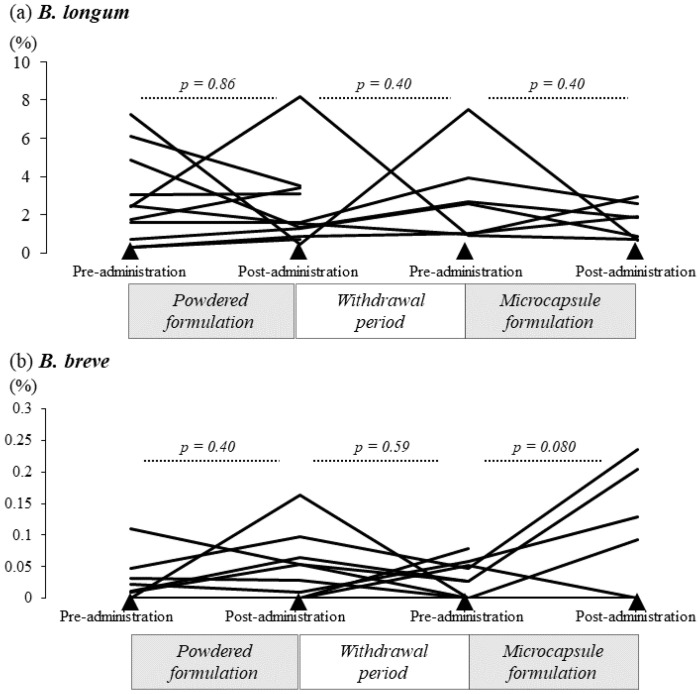
Changes in the relative abundance of *B. longum* or *B. breve* in the gut microbiota after the administration of the powder or acid-resistant microcapsule formulation containing bifidobacteria. (**a**) The relative abundance of *B. longum* in the gut microbiota, calculated from the results of 16S rRNA gene analysis, was not significantly changed after administration of the powder or acid-resistant microcapsule formulation or before or after the withdrawal period. The relative abundance of *B. breve* also did not change significantly before or after administration of the powder formulation or before or after the withdrawal period. (**b**) By contrast, the relative abundance of *B. breve* tended to increase after administration of the microcapsule formulation, although this difference was not statistically significant compared with the relative abundance before administration.

The relative abundance of *B. longum* and *B. breve* was determined by 16S rRNA sequencing before and after administration of the formulations (Figure 5). As shown in Figure 5a, the relative abundance of *B. longum* was not significantly increased after 1 month of powder administration (median 1.51% [IQR = 1.07–3.25]) compared with the value before administration (median 2.41% [IQR = 1.16–3.93%], *p* = 0.86). There was no statistically significant difference in the relative amounts before and after the one-month withdrawal period (*p* = 0.40). Furthermore, the abundance of *B. longum* did not differ before and after microcapsule formulation administration (median 2.57% [IQR = 0.99–3.29%] versus median 1.85% [IQR = 0.77–2.23, *p* = 0.40).

Meanwhile, as shown in Figure 5b, the abundance of *B. breve* was 0.053% (median; IQR = 0.0–0.07) after 1 month of powder formulation administration, compared with 0.011% (median; IQR = 0.0–0.03) before treatment (*p* = 0.40). There was no statistically significant difference in relative abundance before and after the one-month withdrawal period (*p* = 0.59). Conversely, the median relative abundance of *B. breve* tended to increase after microcapsule formulation administration (0.028% [IQR = 0–0.050%] versus 0.093% [IQR = 0–0.167]), although the difference did not reach significance (*p* = 0.080).

#### 3.2.4. Quantification of *B. breve*

**Figure 6:**
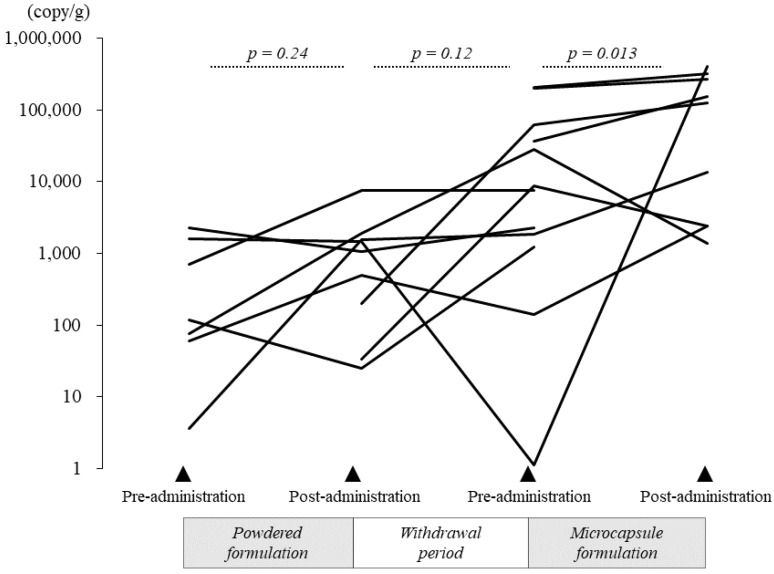
Quantitative changes in *B. breve* after administration of the powder or microcapsule formulation containing bifidobacteria. The graph presents the quantitative changes in the bacterial count of *B. breve* before and after administration of the powder formulation, before and after a one-month withdrawal period, and before and after administration of the acid-resistant microcapsule formulation by quantitative PCR. *B. breve* did not show a statistically significant increase after administration of the powder formulation. However, after administration of the acid-resistant microcapsule formulation, the bacterial count of *B. breve* was significantly higher than the bacterial count before administration.

As only *B. breve* tended to increase in relative abundance after microcapsule formulation administration, as determined by 16S rRNA sequencing (*p* = 0.080), further quantitative analysis of *B. breve* using RT-PCR was performed. The results are presented in Figure 6 (bacterial counts were calculated per gram of stool). After administration of the *B. breve* powder formulation, the median bacterial count slightly increased to 1479.57 copies/g (median; IQR = 769.6–1715.4 copies/g), compared with 116.5 copies/g (median; IQR = 68.1–1163.7 copies/g) before administration, but the difference did not reach statistical significance (*p* = 0.24). The change in the median bacterial count of *B. breve* before and after the one-month withdrawal period was not statistically significant (*p* = 0.12). By contrast, the median bacterial count of *B. breve* was significantly higher after 1 month of microcapsule formulation administration (28,069 copies/g [IQR = 1866.3–62,721.2] before administration versus 153,576.4 copies/g [IQR = 13,433.4–323,813.1] after administration, *p* = 0.013).

#### 3.2.5. Comparison of the Colon Delivery Efficiency of the Powder and Microcapsule Formulations of *B. breve*

The efficiency at which *B. breve* reached the large intestine was compared between the formulations using the following formula: Number of PCR copies (copies/g) × 1000 g (predicted daily defecation volume)/1 billion (calculated bacterial count of daily oral intake of *B. breve* by powder formulation) or 100 million (calculated bacterial count of daily oral intake of *B. breve* by microcapsule formulation).

**Figure 7:**
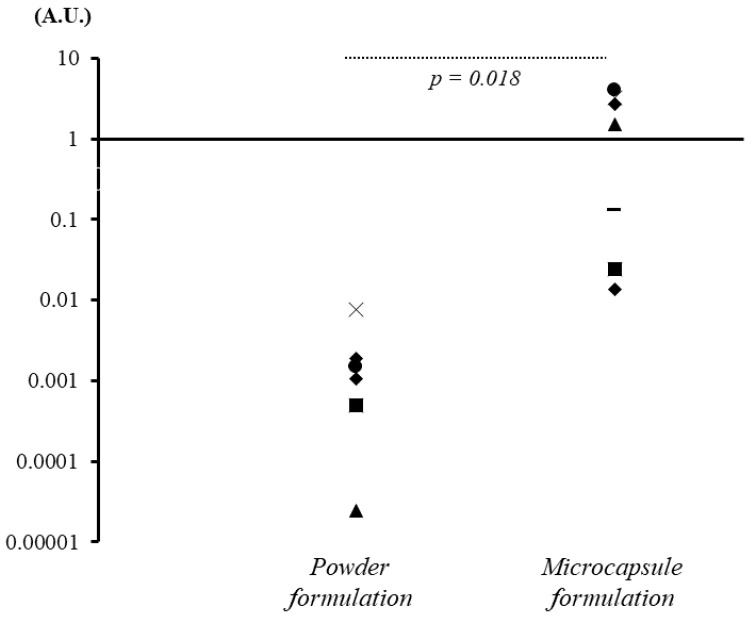
Differences in the efficiency at which *B. breve*-containing powder or acid-resistant microcapsule formulations reached the large intestine after administration. The graphs present the efficiency at which *B. breve* reached the large intestine calculated by quantitative PCR. Compared with the effects of the powder formulation, the acid-resistant microcapsule formulation significantly improved the efficiency at which the bacteria reached the large intestine after administration. Each symbol, such as circle, square, triangle, diamond, bar, and cross, denotes the efficiency data from the individual participants. A.U. (arbitrary unit): For the calculation of the efficiency, the following formula is applied: Number of PCR copies (copies/g) × 1000 g (predicted daily defecation volume)/1 billion (*B. breve* content in powder formulation) or 100 million *(B. breve* content in microcapsule formulation).

Figure 7 presents the results of the colon delivery efficiency of *B. breve*. The microcapsule formulation of *B. breve* was delivered to the large intestine with significantly better efficiency than the powder formulation (1.54 [IQR = 0.079–2.98] versus 0.0015 [IQR = 0.00077–0.0017], respectively, *p* = 0.018).

## 4. Discussion

A previous study confirmed the viability of powdered and capsule probiotic formulations containing bifidobacteria in a validated dynamic in vitro model of the stomach and small intestine, simulating a human adult. The results showed that the survival rate of the capsule formulation was approximately twice that of the powder formulation [24]. Therefore, the administration of a capsule formulation of probiotics is desirable; however, the large size of the capsule formulation makes it difficult for children to swallow. Therefore, we have developed microcapsules containing bifidobacteria of size (2 mm) that can be taken internally, even by toddlers. As shown in Figure 1a, acid-resistant microcapsules are almost spherical in shape with a diameter of 2 mm, differing from the hard or soft capsules that are widely used in general medicine and food products. This structure does not allow external liquids or other substances to penetrate through the membranes. These microcapsules were prepared by the “drop-in-liquid method,” which utilizes interfacial tension to impart properties such as acid resistance and enteric solubility to the membrane. This design enables bifidobacteria, which are vulnerable to stomach acid, to be successfully delivered to the large intestine. After a microcapsule is orally consumed, it passes through the stomach, where the pH is low, and by the time it reaches the intestine, the pH changes to near neutral, which causes the pH-responsive outer skin membrane to dissolve. The microcapsule is then collapsed within the intestinal tract via the physical stimulation of intestinal movement, and living bifidobacteria are delivered [21]. The microcapsules in the present study contained *B. breve* M-16V and *B. longum* BB536. *B. breve* M-16V is a non-motile, non-spore-forming, rod-shaped, anaerobic Gram-positive bacterium discovered in 1963 in the intestines of infants [25]. *B. breve* M-16V is known to reach the human gastrointestinal tract and has strong adhesion activity [26]. Comprehensive safety evaluations, including functional, genomic, and in vivo analyses, have shown that *B. breve* M-16V is a non-pathogenic, non-toxigenic, non-hemolytic, non-antibiotic-resistant probiotic bacterium that contains no plasmids and shows no harmful metabolic activity [26,27,28]. The other component, *B. longum* BB536, was discovered in the intestines of healthy breastfed infants in 1969 [29]. *B. longum* BB536 has been used as a functional food ingredient in a variety of products, including dairy beverages, yogurt, powdered milk, and nutritional supplements, and has been sold in more than 30 countries for over 40 years [29]. *B. longum* BB536 is a Gram-positive, anaerobic, catalase-negative bacillus with an irregular morphology and excellent stability during storage. It is known to be highly viable in probiotic foods until consumption, and it does not contain plasmids and exhibits no harmful metabolic activity [26,27]. An in vivo study was also conducted in which high doses of BB536 were orally administered to healthy 4-week-old mice for 7 days, and it was found that BB536 did not migrate to the blood, liver, spleen, kidney, and mesenteric lymph nodes and did not induce damage to the intestinal surface [28].

In this study, we evaluated whether the microcapsule formulation reached the colon more efficiently than the powder formulation by quantitative PCR using specific primers for *B. breve*. Significantly more *B. breve* specimens were detected in the stool after microcapsule formulation administration than after powder administration. In addition, the microcapsule formulation reached the intestinal tract more efficiently than the powder formulation, as was calculated from the content of *B. breve* present in the stool.

The microcapsule formulation contained *B. longum* in addition to *B. breve*. Despite the high total bacterial content of *B. longum*, there was no increase in *B. longum* after administration. The reasons for these findings were as follows: 1) The results of the present study showed that *B. breve* and *B. longum* have absolutely different proportions in the gut microbiota, with *B. longum* being about 100 times more abundant. Therefore, it is possible that the amount of *B. longum* in the microcapsule could not be detected in the stool as a significant difference even if the amount of *B. longum* was 10 times higher than that of *B. breve*; 2) It has been reported that Gram-positive bacteria must be sufficiently cell-disrupted during the DNA extraction process; otherwise, the extraction sensitivity may be reduced [30]. Both *B. longum* and *B. breve* may have low detection sensitivity because they are Gram-positive bacteria, and this may be the reason why no significant change was observed in the relative abundance of *B. longum* and *B. breve* by16S-rRNA gene analysis using NGS.

This study had four limitations. First, the efficiency at which viable bacteria reached the intestine was not calculated by culturing bifidobacteria extracted from the stool. However, we believed that we could evaluate the efficiency using quantitative PCR and bacterial 16S rRNA gene analysis. Second, the study participants displayed no changes in stool quality or improvement in constipation following *bifidobacterium* administration. The study participants did not include children who originally had constipation, and thus, it is necessary to confirm the efficacy of the oral microcapsule formulation in children with constipation in the future. Third, the number of participants in this study was small. Because this was an interventional study in healthy children, the number of participants was small as a result of ethical concerns. However, we were able to demonstrate the efficacy of microcapsules containing bifidobacteria, and we are considering conducting a larger-scale trial in the future. Fourth, the present study was designed based on the report by Saxelin et al. [31] that proposed that the effect of the administered probiotic would disappear from the subject’s colon after a one-month withdrawal period following the end of oral administration. However, as shown in Figure 6, a statistically significant decrease in *B. breve* was not observed during the withdrawal period, so the “priming effect” cannot be ruled out. Therefore, based on the results of this pilot study, we plan to incorporate a parallel design in our next large-scale intervention study.

In conclusion, we confirmed that the developed acid-resistant microcapsule formulation can efficiently deliver *B. breve* to the intestine. *B. breve* is classified as an infantile form of HRB, and acid-resistant microcapsules that can efficiently deliver it to the intestine as a probiotic formulation for children have tremendous advantages. In addition, considering the size of the microcapsule, we believe it will be easy to add the microcapsule to food products such as yogurt in the future.

## Figures and Tables

**Table 1:** Details of the study participants.

	Powder Formulation	Microcapsule Formulation	*p*-Value
Adherence (%)	100 (92.9–100)	100 (96.6–100)	0.49
Frequency of defecation per week (pre/post)	5.0 (4.5–6.0)/5.75 (5.0–6.0)	5.75 (5.0–6.0)/ 3.25 (2.75–5.75)	0.35/0.18 ^‡^
BSFS score ^†^ (pre/post)	4.0 (3.0–4.0)/4.0 (3.0–4.0)	4.0 (3.0–4.0)/4.0 (4.0–4.0)	0.56/0.32 ^‡^

^†^ BSFS, Bristol Stool Form Scale; ^‡^ Statistical comparison between before and after administration.

## Data Availability

The datasets generated and analyzed during the current study are available from the corresponding authors upon reasonable request.
